# Alternative sigma factor B (σ^B^) and catalase enzyme contribute to *Staphylococcus epidermidis* biofilm’s tolerance against physico-chemical disinfection

**DOI:** 10.1038/s41598-019-41797-8

**Published:** 2019-03-29

**Authors:** Charles Ochieng’ Olwal, Paul Oyieng’ Ang’ienda, Daniel Otieno Ochiel

**Affiliations:** Department of Zoology, Maseno University, 333-40105 Maseno, Kenya

## Abstract

*Staphylococcus epidermidis* is the predominant cause of recalcitrant biofilm-associated infections, which are often highly resistant to antibiotics. Thus, the use of physico-chemical agents for disinfection offers a more effective approach to the control of *S. epidermidis* biofilm infections. However, the underlying tolerance mechanisms employed by *S. epidermidis* biofilm against these physico-chemical disinfectants remain largely unknown. The expression of a σ^B^-dependent gene, alkaline shock protein 23 (*asp23*) and catalase activity by *S. epidermidis* biofilm and planktonic cells exposed to heat (50 °C), 0.8 M sodium chloride (NaCl), 5 mM sodium hypochlorite (NaOCl) or 50 μM hydrogen peroxide (H_2_O_2_) for 60 minutes were compared. Significantly higher *asp23* expression levels were observed in biofilms exposed to 50 °C, 5 mM NaOCl or 50 μM H_2_O_2_ compared to the corresponding planktonic cells (*p* < 0.05). Conversely, *asp23* expression levels in biofilm and planktonic cells exposed to 0.8 M NaCl were not significantly different (*p* > 0.05). Further, biofilms exposed to 50 °C, 0.8 M NaCl, 5 mM NaOCl or 50 μM H_2_O_2_ exhibited significantly higher catalase activity than the planktonic cells (*p* < 0.05). These results suggest that activities of σ^B^ and catalase may be involved in the tolerance of *S. epidermidis* biofilm against physico-chemical disinfection.

## Introduction

*Staphylococcus epidermidis* biofilm is the predominant cause of primary bacteremia^1^ and medical implant device infections^2^. *S*. *epidermidis* biofilm is highly resistant to antibiotics and host immunity^1^. Hence, treatment of *S. epidermidis* biofilm-based infections often entails removal and replacement of the infected device, increasing morbidity and cost^1^. Effective control of *S. epidermidis* biofilm-associated infections is further hampered by the relatively high tendency of bacteria to develop resistance to antibiotics^3^. Consequently, the use of effective physico-chemical disinfection procedures to control biofilm-forming bacteria, such as *S. epidermidis*, is necessary^4^ in both domestic and healthcare settings, where bacterial biofilms are frequently encountered^5,6^. Nevertheless, the underlying tolerance mechanisms employed by *S. epidermidis* biofilm against physico-chemical disinfection remain largely unknown.

Multiple mechanisms have been proposed to account for the relatively high tolerance of biofilms to antimicrobials. These include, reduced diffusion of antimicrobials through the biofilm matrix^7^, neutralization of the antimicrobials by the biofilm matrix components^8^, physiological heterogeneity conferred by the three-dimensional biofilm structure^9^, higher expression of specific protective molecules^10^, and the presence of a subpopulation of highly resistant cells (persisters)^9^. However, these mechanisms only partially account for the high tolerance of biofilms against few antibiotics e.g. ciprofloxacin^2,7,11^, but not tolerance against commonly used physico-chemical disinfectants, such as heat, NaCl, NaOCl or H_2_O_2_.

The alternative factor B (σ^B^), a sub-unit of RNA polymerase^12^, has been implicated in the tolerance of planktonic forms of different bacterial species against several stress agents, including heat^13–16^, osmotic stress^15–18^, H_2_O_2_^13,14^ or antibiotics^19,20^ exposure. Although σ^B^ is essential in *S. epidermidis* biofilm formation, maturation and stability^21^, its role in the tolerance of *S. epidermidis* biofilm against heat, NaCl, NaOCl or H_2_O_2_ exposure has not been determined. The *sigma B* gene expression is dependent on both a σ^A^ and σ^B^ promoter^22,23^. On the contrary, alkaline shock protein 23 (*asp23*) gene expression is almost exclusively transcribed from σ^B^-dependent promoters, making it a good marker for σ^B^ activity in *S. epidermidis*^22,24^.

Catalase enzyme has been linked with the survival of planktonic forms of different bacterial species against osmotic stress^25,26^, H_2_O_2_^26,27^ and nitric oxide^28^ exposure. Despite *S. epidermidis* being a catalase-producing bacterium^29^, the role of catalase in the tolerance of *S. epidermidis* biofilm against heat, NaCl, NaOCl or H_2_O_2_-exposure has not been fully investigated.

Therefore, to better understand the mechanisms of tolerance of *S. epidermidis* biofilm against physico-chemical disinfection, the present study compared the expression of a σ^B^-dependent gene, *asp23* and catalase activity of *S. epidermidis* biofilm and planktonic cells in response to heat, NaCl, NaOCl or H_2_O_2_ exposure.

## Results

### Optimal concentrations of physico-chemical disinfectants for analysis of *S. epidermidis* biofilm tolerance mechanisms

**Figure 1:**
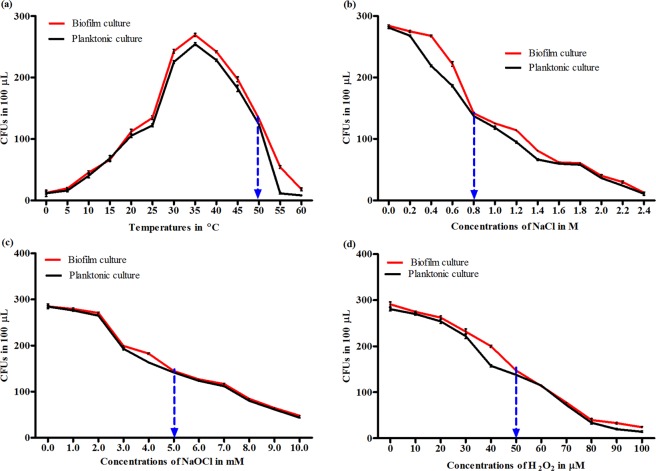
The growth of *S. epidermidis* cells exposed to increasing physico-chemical disinfectant concentrations. The growth curves for a single mixture of *S. epidermidis* biofilm or planktonic culture subjected to increasing temperatures (**a**) or concentrations of NaCl (**b**), NaOCl (**c**) or H_2_O_2_ (**d**) for 60 minutes. The plots at each physico-chemical disinfectant concentration depict the mean ± standard deviation of three independent experiments with three technical replicates. The blue dotted arrow represents the optimal temperature/concentration at which both *S. epidermidis* biofilm and planktonic cells were considerably stressed (growth reduced by almost 2-fold with reference to the highest CFU value).

When subjected to increasing temperatures/concentrations of the physico-chemical disinfectants for 60 minutes, the *S. epidermidis* biofilm and planktonic cells were considerably stressed (growth reduced by almost 2-fold with reference to the highest CFU values) at 50 °C, 0.8 M NaCl, 5 mM NaOCl or 50 μM H_2_O_2_ (Fig. 1). Thus, the above temperature/concentrations of the physico-chemical disinfectants were considered optimal for analysis of *asp23* expression and catalase activity in *S. epidermidis* biofilm and planktonic cells.

### *Asp23* expression is upregulated more in heat-exposed biofilm compared to planktonic cells

**Figure 2:**
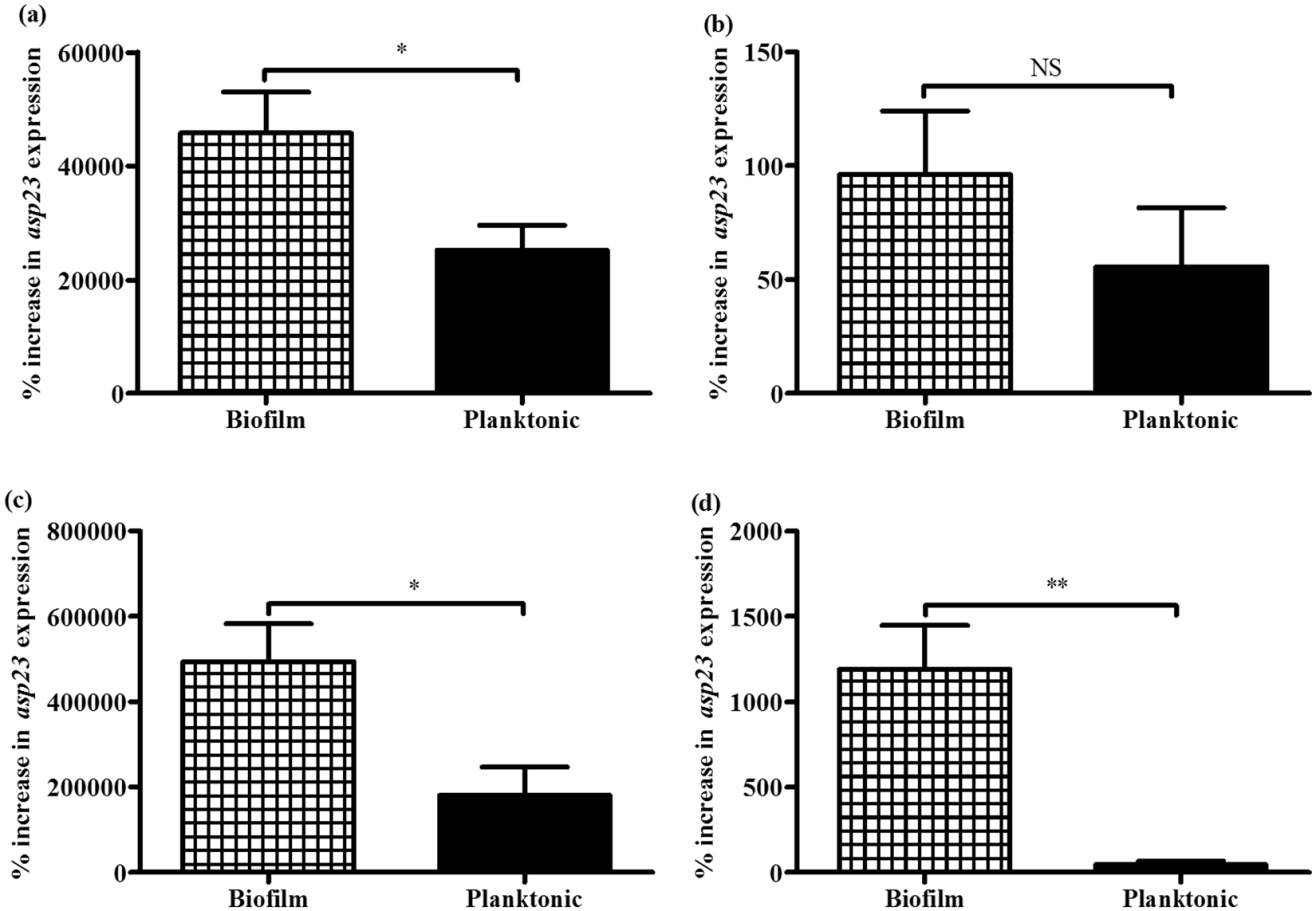
Effects of physico-chemical disinfectant-exposure on *asp23* expression by *S. epidermidis* cells. The percent increase in *asp23* expression of pairs of *S. epidermidis* biofilm (n = 10) and planktonic (n = 10) samples exposed to 50 °C (**a**), 0.8 M NaCl (**b**), 5 mM NaOCl (**c**) or 50 μM H_2_O_2_ (**d**) for 60 minutes. For each sample, three repeats of *asp23* expression measurements were performed. Bars represent the mean ± SEM. Statistical difference between biofilm and planktonic cells was evaluated using paired *t*-test (NS, *p* > 0.05; ^*^p < 0.05; ^**^p < 0.01).

The *S*. *epidermidis* biofilm cells exposed to 50 °C temperature for 60 minutes had a significantly higher expression of a σ^B^-dependent gene, *asp23* compared to the corresponding planktonic cells (*p* = 0.0259; Fig. 2a). Further, the *S. epidermidis* biofilm or planktonic cells exposed to 50 °C temperature for 60 minutes exhibited significantly increased *asp23* expressions than their respective cells exposed to 25 °C temperature (controls) (*p* < 0.0001; Table 2). Taken together, these results indicated that 50 °C-exposure enhances σ^B^ activity in both *S. epidermidis* biofilm and planktonic cells, but with significantly higher activity levels in the biofilm cells.

**Table 1:** Primers used for qPCR amplification.

Target gene	Set	Sequence (5′–3′)	Reference
***S. epidermidis***			
*asp23*	Forward	CAGCAGCTTGTTTTTCTCCA	^21^
	Reverse	CATGAAAGGTGGCTTCACAG	
16S rRNA	Forward	GGGCTACACACGTGCTACAA	^44^
	Reverse	GTACAAGACCCGGGAACGTA	
***S. aureus*** **ATCC 29213**			
*asp23*	Forward	TCGCTGCACGTGAAGTTAAA	^53^
	Reverse	CAGCAGCTTGTTTTTCACCA	
16S rRNA	Forward	GTAGGTGGCAAGCGTTATCC	^54^
	Reverse	CGCACATCAGCGTCAG

**Table 2:** Effect of physico-chemical disinfectant-exposure on *asp23* expressions by *S*. *epidermidis* cells.

Type of cell	Mean ± SEM of *asp23* expressions in response to physico-chemical disinfectants exposures			
	Heat	NaCl	NaOCl	H_2_O_2_
**Biofilm (n = 10)**				
Unexposed	0.0178 ± 0.0021	0.0062 ± 0.0012	0.0286 ± 0.0051	0.0078 ± 0.0010
Exposed	7.4780 ± 0.9350	0.0127 ± 0.0032	112.11 ± 16.720	0.0869 ± 0.0161
	*p* < **0.0001**	*p* = **0.0203**	*p* < **0.0001**	*p* = **0.0002**
**Planktonic (n = 10)**				
Unexposed	0.0204 ± 0.0017	0.0052 ± 0.0011	0.0425 ± 0.0085	0.0141 ± 0.0019
Exposed	4.7510 ± 0.6449	0.0081 ± 0.0028	56.474 ± 16.37	0.0220 ± 0.0055
	*p* < **0.0001**	*p* = 0.1831	*p* = **0.0073**	*p* = 0.0725

The *asp23* expression levels of *S. epidermidis* biofilm and planktonic cells exposed to 50 °C, 0.8 M NaCl, 5 mM NaOCl or 50 μM H_2_O_2_ for 60 minutes and the unexposed controls. For each sample, three repeats of *asp23* expression measurements were performed. Boldface represent a statistically significant difference between the physico-chemical disinfectant-exposed cells and the unexposed controls as determined by paired-*t* test (*p* < 0.05).

### NaCl exposure enhances *asp23* expression in biofilm cells, but not in planktonic cells

When exposed to 0.8 M NaCl for 60 minutes, the expression levels of *asp23* between *S. epidermidis* biofilm and planktonic cells were not significantly different (*p* = 0.4029; Fig. 2b). However, *ap23* expression by 0.8 M NaCl-treated *S. epidermidis* biofilm cells was significantly higher compared to that of the untreated controls (*p* = 0.0203; Table 2). These findings indicated that 0.8 M NaCl exposure specifically enhances σ^B^ activity in *S. epidermidis* biofilm cells, but not in the corresponding planktonic cells.

### NaOCl or H_2_O_2_ exposure elevates *asp23* expression to higher extent in biofilm cells than in planktonic cells

The *S. epidermidis* biofilm or planktonic cells treated with 5 mM NaOCl for 60 minutes exhibited significantly increased *asp23* expressions than their respective unexposed controls (*p* < 0.05; Table 2). Further analysis showed that *asp23* expression was significantly higher in *S. epidermidis* biofilm cells compared to the corresponding planktonic cells upon exposure to 5 mM NaOCl for 60 minutes (*p* = 0.0109; Fig. 2c). Moreover, *S. epidermidis* biofilm cells exhibited significantly elevated *asp23* expression levels compared to the corresponding planktonic cells upon exposure to 50 μM H_2_O_2_ for 60 minutes (*p* = 0.0020; Fig. 2d). Although the *asp23* expressions by *S*. *epidermidis* planktonic cells upon 50 μM H_2_O_2_-exposure was not significantly different compared to their respective unexposed controls (*p* = 0.0725; Table 2), the *ap23* expression by 50 μM H_2_O_2_-treated *S. epidermidis* biofilm cells was significantly higher than in the unexposed controls (*p* = 0.0002; Table 2). Taken together, these results indicated that 5 mM NaOCl and 50 μM H_2_O_2_-exposure significantly enhances σ^B^ activity in *S*. *epidermidis* biofilm cells than in the corresponding planktonic cells.

### Heat or NaCl-exposed biofilm cells produce higher amounts of catalase than the planktonic cells

**Figure 3:**
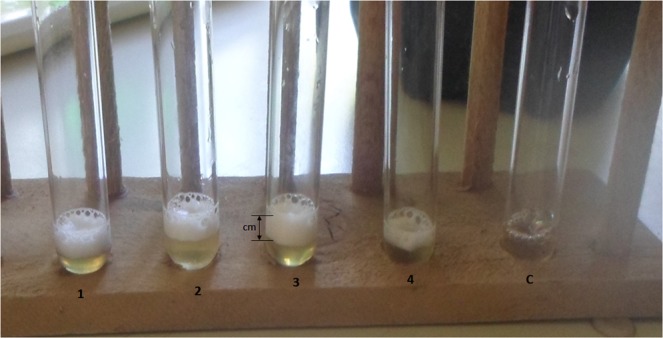
Images of the height of foam. The test tubes show the foam formed during H_2_O_2_-catalase reaction. Test tubes that formed well spread foam as shown in test tubes labeled 1–4 were measured in cm as illustrated in test tube labeled 3 (readings were taken at the bottom of the lower and upper meniscus). Test tubes that did not form a well spread foam as presented in test tube labeled C were not quantified.

In this study, using a simple visual assay, we quantified catalase activity by measuring the trapped oxygen (O_2_) gas, which is visualized as foam. The O_2_ gas generated during the H_2_O_2_-catalase reaction in the test tubes and how the measurements were done is shown in Fig. 3.

**Figure 4:**
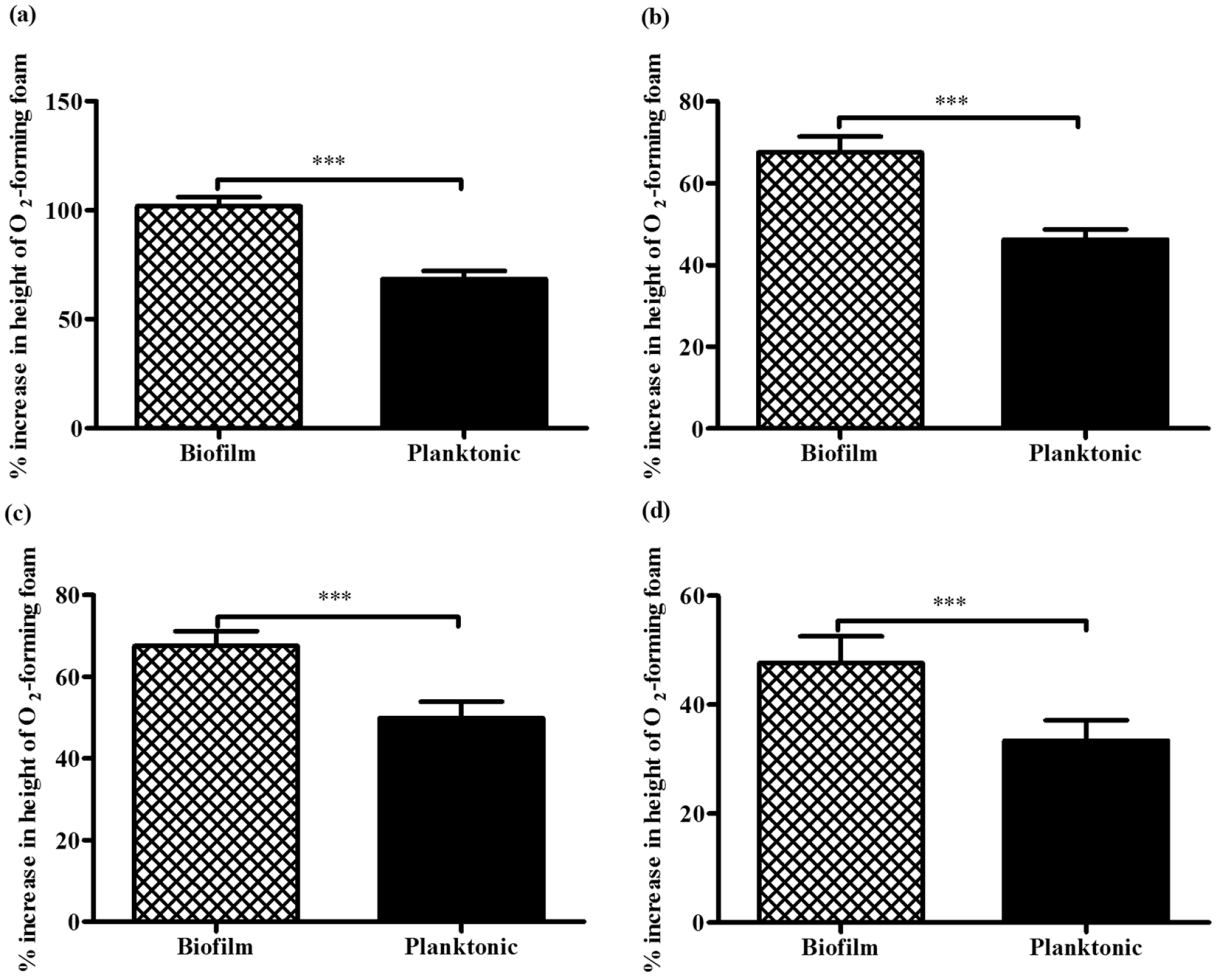
Effects of physico-chemical disinfectant-exposure on catalase release by *S. epidermidis* cells. The percent increase in height of O_2_-forming foam for pairs of *S. epidermidis* biofilm (n = 62) and planktonic (n = 62) samples exposed to 50 °C (**a**), 0.8 M NaCl (**b**), 5 mM NaOCl (**c**) or 50 μM H_2_O_2_ (**d**) for 60 minutes. For each sample, three repeats of catalase activity measurements were performed. Bars represent the mean ± SEM. Statistical difference between biofilm and planktonic cells was determined by paired *t*-test (^*^p < 0.05; ^***^p < 0.001).

The *S. epidermidis* biofilm cells exposed to 50 °C for 60 minutes had a significantly higher height of foam compared to the corresponding planktonic cells (*p* < 0.0001; Fig. 4a). Moreover, a significant increase in height of foam was observed in *S. epidermidis* biofilm cells exposed to 0.8 M NaCl for 60 minutes compared to the analogous planktonic cells (*p* < 0.0001; Fig. 4b). Further, *S. epidermidis* biofilm or planktonic cells exposed to 50 °C or 0.8 M NaCl for 60 minutes exhibited significantly higher heights of foam than their respective unexposed controls (*p* < 0.0001; Table 3). Taken together, these findings implied that both *S. epidermidis* biofilm and planktonic cells enhance catalase release in response to 50 °C or 0.8 M NaCl exposure, but with significantly higher release by the biofilm cells.

**Table 3:** Impact of physico-chemical disinfectant-exposure on catalase activity in *S*. *epidermidis* cells.

Type of cell	Mean ± SEM of height of O_2_-forming foam in response to physico-chemical disinfectant exposure			
	Heat	NaCl	NaOCl	H_2_O_2_
**Biofilm (n = 62)**				
Unexposed	0.2258 ± 0.0052	0.2226 ± 0.0064	0.2242 ± 0.0053	0.2226 ± 0.0064
Exposed	0.4479 ± 0.0071	0.3640 ± 0.0059	0.3672 ± 0.0068	0.3151 ± 0.0067
	*p* < 0.0001	*p* < 0.0001	*p* < 0.0001	*p* < 0.0001
**Planktonic (n = 62)**				
Unexposed	0.2505 ± 0.0056	0.2516 ± 0.0057	0.2511 ± 0.0057	0.2522 ± 0.0061
Exposed	0.4129 ± 0.0068	0.3602 ± 0.0058	0.3651 ± 0.0067	0.3258 ± 0.0070
	*p* < 0.0001	*p* < 0.0001	*p* < 0.0001	*p* < 0.0001

The height of O_2_-forming foam of *S. epidermidis* biofilm and planktonic cells exposed to 50 °C, 0.8 M NaCl, 5 mM NaOCl or 50 μM H_2_O_2_ for 60 minutes and the unexposed controls. Statistical differences between physico-chemical disinfectant-exposed cells and the unexposed controls were determined using paired *t*-test (all the differences were significant; *p* < 0.05).

### NaOCl or H_2_O_2_-exposed biofilm cells release more catalase than the planktonic cells

The *S. epidermidis* biofilm cells subjected to 5 mM NaOCl for 60 minutes had a significantly higher height of foam compared to the corresponding planktonic cells (*p* < 0.0001; Fig. 4c). Similarly, a significantly higher height of foam was observed in *S. epidermidis* biofilm cells exposed to 50 μM H_2_O_2_ for 60 minutes compared to the planktonic cells (*p* < 0.0001; Fig. 4d). Further, *S. epidermidis* biofilm or planktonic cells exposed to 5 mM NaOCl or 50 μM H_2_O_2_ for 60 minutes exhibited significantly higher heights of foam than their respective unexposed controls (*p* < 0.0001; Table 3). Taken together, these results indicated that 5 mM NaOCl or 50 μM H_2_O_2_-exposure stimulates catalase release by *S. epidermidis* biofilm and planktonic cells, with significantly higher release by the biofilm cells.

## Discussion

The mechanisms underlying the tolerance of *S. epidermidis* biofilm against physico-chemical disinfection remain largely unknown. Therefore, in this study, the activities of σ^B^ and catalase were evaluated as potential tolerance mechanisms employed by *S. epidermidis* biofilm against heat, NaCl, NaOCl or H_2_O_2_-exposure.

Results presented showed that σ^B^ activity is enhanced in both *S. epidermidis* biofilm and planktonic cells in response to 50 °C-exposure, but with significantly higher activities in the biofilm cells. The present finding is in agreement with previous reports on *B*. *cereus*^15^ and *B*. *subtilis*^16^. However, the previous studies only focused on the planktonic forms of the *Bacillus* species. Microbial metabolic activities are temperature-dependent^30^. Considering that σ^B^ regulates bacterial metabolism^31^ and that planktonic cells have higher metabolic activity than biofilm^32^, it was expected that planktonic cells would have a higher σ^B^ activity than the corresponding biofilm cells. However, the observed nearly 2-fold higher σ^B^ activity by *S*. *epidermidis* biofilm compared to the planktonic cells suggests that the tolerance of biofilm against heat exposure might be more dependent on σ^B^ activity.

Further, we showed that 0.8 M NaCl-exposure enhances σ^B^ activity in the *S. epidermidis* biofilm cells, but not in the planktonic cells. Our finding that σ^B^ activities between 0.8 M NaCl-treated planktonic cells and their untreated controls were not statistically different contradicts previous reports on different bacterial species^14,15,17,18^. Considering that NaCl-exposure response regulatory mechanisms in different bacterial species may not follow common patterns^33^, the discrepancy between the present and previous outcomes could be attributed to the different regulatory patterns in planktonic cells of *S. epidermidis* and the previously studied bacterial species. Although we did not observe a significant difference in σ^B^ activity between NaCl-exposed *S. epidermidis* planktonic cells and the unexposed controls, some low level of σ^B^ activity was detected. Perhaps the low levels of σ^B^ activity detected in the planktonic cells could be as a result of the basal σ^B^ activity^22^, and not necessarily as a NaCl-exposure response mechanism. A possible explanation for the observed significantly elevated σ^B^ activity levels in biofilm cells, but not in planktonic cells is that tolerance of *S. epidermidis* biofilm against NaCl-exposure might be more specifically dependent on σ^B^ activity.

Further, our data showed that 5 mM NaOCl-exposure upregulates σ^B^ activity in both *S. epidermidis* biofilm and planktonic cells, but with significantly higher activities in the biofilm cells. So far, there are no direct reports on the effect of NaOCl exposure on σ^B^ activity. NaOCl-exposure exerts its anti-bacterial effects by targeting several metabolic processes, such as DNA synthesis, adenosine triphosphate synthesis etc.^34^. The same metabolic processes are regulated by σ^B^ activity^31^. Considering that planktonic cells have higher metabolic activity than the corresponding biofilm^32^, planktonic cells should have exhibited higher σ^B^ activity in response to NaOCl exposure. However, we observed the opposite, suggesting that tolerance of *S. epidermidis* biofilm against NaOCl exposure is more dependent on the σ^B^ activity.

Furthermore, we revealed that 50 μM H_2_O_2_ exposure enhances σ^B^ activity in *S. epidermidis* biofilm cells, but not in the planktonic cells. The present findings concur with a previous report, showing that 50 μM H_2_O_2_ has a limited effect on σ^B^ expression by *B*. *cereus* cells^15^. Moreover, the present finding agrees with a previous report in which 60 μM H_2_O_2_ was shown not to affect σ^B^ expression in *S. aureus* cells^14^. However, the previous reports only focused on the planktonic forms of *B. cereus* and *S. aureus*. It is probable that no σ^B^ expressions were detected in the 60 μM H_2_O_2_-exposed to *S. aureus*^14^ because the study employed northern blot analysis, which is insensitive and only detects large gene expressions^35^. Taking into the findings of the present and previous studies, it appears that σ^B^ activity by planktonic cells is largely independent of H_2_O_2_-exposure. The low levels of σ^B^ activity detactable in planktonic cells in the present and previous studies could be probably be related to the basal σ^B^ activity^22^, but not necessarily as a H_2_O_2_-stress response mechanism. A more plausible explanation for the observed significantly higher σ^B^ activities in the biofilm cells, but not in planktonic cells is that σ^B^ has a more significant role in the *S. epidermidis* biofilm’s tolerance against H_2_O_2_-exposure.

Further results of this study demonstrated that 50 °C-exposure enhances catalase release by *S. epidermidis* biofilm and planktonic cells, but with higher release by the biofilm cells. The present finding is consistent with two previous reports on a fungus *Aspergillus nidulans*^36^ and *Rhodobacter sphaeroides*^37^. However, the previous reports only focused on the planktonic forms of the two organisms. Taking into account that temperatures above 37 °C increases the porosity of *S. epidermidis* biofilm^38^, a probable explanation for the higher catalase release by the biofilms could be related to the increased porosity of the *S. epidermidis* biofilm by the 50 °C-exposure thus availing more catalase for quantification. Alternatively, catalase release is a metabolic process, which is expected to be higher in planktonic cells compared to the biofilm^32^; however, *S. epidermidis* biofilm exhibited higher catalase activity in response to 50 °C-exposure suggesting a more important role of catalase in biofilm’s tolerance against heat-exposure.

Further, we showed that 0.8 M NaCl exposure stimulates catalase release by both *S. epidermidis* biofilm and planktonic cells, but with significantly higher release by the biofilm cells. Our observation that 0.8 M NaCl-treated planktonic cells released higher catalase than the untreated controls is in line with previous reports on different bacterial species^25,26^. Additional analyses demonstrated that 5 mM NaOCl or 50 μM H_2_O_2_-exposure enhances catalase release by *S. epidermidis* biofilm and planktonic cells, but with significantly more production by the biofilm cells. Our observations that NaOCl or H_2_O_2_-exposed planktonic cells produce significantly higher catalase than the unexposed controls are in agreement with previous reports on different bacterial species^37,39^. However, a contradicting observation has been reported for *P. aeruginosa* exposed to H_2_O_2_^40,41^. The differences in the exposure durations prior to catalase measurements could be responsible for the observed discrepancy between the results of the present and the two previous studies. From the present findings, it appears that catalase activity is involved in both *S. epidermidis* biofilm and planktonic cells’ tolerance against NaCl, NaOCl or H_2_O_2_-exposure. However, considering that catalase production is a metabolic activity, we expected planktonic cells to show significantly higher catalase activity than the biofilm^32^ per unit viable cells. We, instead, observed the opposite, suggesting that catalase might be involved to a greater extent in the tolerance of *S. epidermidis* biofilm against NaCl, NaOCl or H_2_O_2_-exposure.

In conclusion, we observed that 50 °C, 0.8 M NaCl, 5 mM NaOCl or 50 μM H_2_O_2_-exposed *S. epidermidis* biofilm cells significantly enhance σ^B^ and catalase activities more than the corresponding planktonic cells, suggesting that both σ^B^ and catalase activities might be having a greater contribution in the tolerance of *S. epidermidis* biofilm against the physico-chemical disinfectants than in the planktonic cells. Therefore, σ^B^ and /or catalase could be explored further as promising targets for the development of more potent anti-staphylococcal biofilm eradication approaches. Nevertheless, further studies incorporating σ^B^ and catalase mutants or promoter reporters are required to reach a more definite conclusion regarding the dependence of *S. epidermidis* biofilm on the activities of σ^B^ and /or catalase enzyme for survival against the physico-chemical disinfectants.

## Methods

### Sample collection, bacterial isolates and growth conditions

Skin swab samples were collected from outpatients at Kisumu County Referral Hospital (KCRH), Kenya in accordance with relevant guidelines and regulations and research approved by the Maseno University Ethics Review Committee (Reference number: MSU/DRPI/MUERC/000187/15). Further, permission to recruit outpatients was granted by the KCRH. Written informed consent was obtained from participants before recruitment. Collection of the skin swabs and *S. epidermidis* isolation on mannitol salt agar (HiMedia Laboratories Pvt. Limited, Nashik, India) were conducted as described elsewhere^42^. The *S. epidermidis* isolates were further identified using routine microbiologic methods namely, Gram staining, catalase, coagulase and novobiocin sensitivity tests and grown on tryptic soy agar (TSA; HiMedia Laboratories Pvt. Limited, Mumbai, India) at 37 °C overnight. *Staphylococcus aureus* American Type Culture Collection (ATCC) 29213, a good former of mature biofilm within 24 hours^43^, was used as a reference control strain. In this study, analogous planktonic cells served as control samples.

Biofilm was formed as previously described^44^ with few modifications. Briefly, a single *S. epidermidis* colony, from a TSA plate, was inoculated into 2 mL of tryptic soy broth (TSB; HiMedia Laboratories Pvt. Limited, Mumbai, India) and incubated at 37 °C with shaking at 120 revolutions per min (rpm) overnight. To form biofilm culture, 100 µL of the overnight culture adjusted to ~1 × 10^9^ colony-forming units (CFU)/mL was simultaneously inoculated into two polystyrene tubes containing 10 mL of fresh TSB supplemented with 1% glucose (Unilab Limited, Nairobi, Kenya), to enhance biofilm formation, and incubated at 37 °C with shaking at 120 rpm for 24 hours. Then, the spent medium in one of the tubes was removed and the biofilm was rinsed twice with 200 μL of 0.9% NaCl. Biofilm formation on the tube was verified by a qualitative tube method biofilm assay with 0.1% crystal violet staining as described in details elsewhere^45^. In case of strong biofilm formation in the first tube, the spent medium in the parallel second tube was carefully removed, and the biofilm was washed twice with 200 μL of 0.9% NaCl. Then, 1 mL of 0.9% NaCl was added to the tube and vortexed for 2 minutes to detach the biofilm cells (vortexing detaches *S. epidermidis* biofilm from surfaces^46,47^). The detached biofilm cell suspension was centrifuged at 10,000 rpm, 4 °C for 10 minutes. To grow planktonic cells, 100 µL of the overnight culture adjusted to ~1 × 10^9^ CFU/mL was inoculated into 10 mL of fresh TSB in a polystyrene tube and incubated at 37 °C with shaking at 120 rpm for 18 hours. Then, the bacterial cells in suspension were centrifuged at 10,000 rpm, 4 °C for 10 minutes. The biofilm or planktonic cell pellets were suspended in 0.9% NaCl and adjusted to ~1 × 10^9^ CFU/mL.

### Optimal concentrations of physico-chemical disinfectants for analysis of the tolerance mechanisms

To minimize variability between samples, the optimal temperature/concentration of each of the physico-chemical disinfectants was determined using a single mixture of *S. epidermidis* biofilm or planktonic culture as previously described^42^ with some modifications. Briefly, 150 μL of ~1 × 10^9^ CFU/mL of a single mixture of *S*. *epidermidis* biofilm or planktonic culture (prepared by mixing equivalent amount i.e. 150 μL of ~1 × 10^9^ CFU/mL of *S. epidermidis* biofilm or planktonic culture drawn from six random biofilm samples or corresponding planktonic samples) was inoculated into 1.5 mL of increasing concentrations of NaCl, NaOCl (Supersleek, Nairobi, Kenya) or H_2_O_2_ (RFCL Limited, New Delhi, India) and exposed for 60 minutes. For heat exposure, tubes containing 1.5 mL of sterile distilled water were inoculated with 150 μL of ~1 × 10^9^ CFU/mL of the single mixture of *S*. *epidermidis* biofilm or planktonic culture and exposed to increasing temperatures in a water bath for 60 minutes. The NaOCl and H_2_O_2_-exposed cultures were neutralized by 200 μL of 0.1% sodium thiosulphate. Whereas, NaCl and heat-exposed cultures were neutralized by 200 μL of sterile distilled water at 4 °C. To collect biofilm cells, the bacteria in suspension were discarded and the biofilm was gently rinsed once with 200 μL of 0.9% NaCl. One mL of 0.9% NaCl was added to the biofilm, vortexed for 2 minutes then centrifuged at 9,000 rpm for 8 minutes. Planktonic cells were collected by centrifuging the bacteria in suspension at 9,000 rpm for 8 minutes. The biofilm or planktonic cell pellets were suspended in 1 mL of sterile distilled water and CFUs enumerated on TSA as described elsewhere^42^. At each temperature/concentration of the physico-chemical disinfectant, three experiments were performed with three technical replicates. In this study, 50 °C, 0.8 M NaCl, 5 mM NaOCl or 50 μM H_2_O_2_ induced considerable stress to the biofilm and planktonic cells hence, used in the subsequent procedure.

### Exposure of biofilm and planktonic cells to optimal physico-chemical disinfectants

*S*. *epidermidis* biofilm (n = 62) and planktonic (n = 62) samples were exposed to the optimal temperature/concentration of the disinfectants as previously described^48^ with some modifications. Briefly, 200 μL of ~1 × 10^9^ CFU/mL of biofilm or planktonic suspension were inoculated into 1100 μL of TSB adjusted to 0.8 M NaCl, 5 mM NaOCl or 50 μM H_2_O_2_, vortexed for 2 minutes and incubated at 37 °C with shaking at 80 rpm for 60 minutes. For heat exposure, 200 μL of biofilm or planktonic suspension adjusted to ~1 × 10^9^ CFU/mL were inoculated into 1100 μL of TSB alone and transferred to a water bath at 50 °C for 60 minutes. The effects of the disinfectants were neutralized as in the preceding section. Untreated controls were set up by inoculating 200 μL of biofilm or planktonic suspension adjusted to ~1 × 10^9^ CFU/mL into 1100 μL of TSB alone and incubated at 37 °C for 60 minutes. A similar set up incubated at 25 °C for 60 minutes served as a control for heat exposure. After incubation, the biofilm or planktonic cells were collected as described in the preceding section. For gene expression analysis, the biofilm or planktonic cells were suspended in 0.9% NaCl, adjusted to ~1 × 10^9^ CFU/mL and immediately transferred into an equal volume of a 1:1 mixture of ice-cold acetone and ethanol, then kept at −80 °C for at least 20 minutes. For catalase activity analysis, viable biofilm and planktonic cells were enumerated in duplicate on TSA as described elsewhere^46^ and adjusted to 1 × 10^7^ cells/mL.

### Quantification of *asp23* expression

Total RNA was isolated from the disinfectant-exposed *S*. *epidermidis* biofilm (n = 10) and planktonic (n = 10) samples and the unexposed controls as previously described^49^. Genomic DNA was degraded using DNase I (New England Biolabs, Ipswich, England) following the manufacturer’s instructions. The purity and concentration of RNA were determined as previously described^50^. RNA samples with an OD_280_/OD_260_ ratio between 1.8 and 2.2 were used for complementary DNA (cDNA) synthesis. Two μg of RNA was reverse transcribed into cDNA using ProtoScript^®^ First Strand cDNA Synthesis kit (New England Biolabs, Ipswich, England) following the manufacturer’s instructions. Quantitative-PCR (qPCR) was performed on a Rotor-Gene Q real-time thermal cycler (Qiagen, Hilden, Germany) as previously described^51^ with few modifications. Briefly, each 20 μL of qPCR reaction mixture contained 10 μL of Lunar Universal qPCR mastermix (New England Biolabs, Ipswich, England), 0.5 μL each of the forward and reverse primers (Inqaba Biotechnical, Pretoria, South Africa) specific for 16S rRNA (reference) and *asp23* (target) genes (Table 1), 7 μL of nuclease-free water and 2 μL of cDNA template. The thermal cycling conditions were as follows: 1 minute at 95 °C, followed by 45 cycles of 15 seconds at 95 °C, 30 seconds at 60 °C. A control lacking the M-MuLV enzyme mix was included in each reaction. The *asp23* expression level was determined using the Efficiency^ΔCt^ method, where, ΔCt = Ct (reference gene)−Ct (target gene)^50^ for the disinfectant-exposed samples and their unexposed controls. All primers had an efficiency of ~100%; hence, the real efficiency i.e. 2 was substituted in the Efficiency^ΔCt^ formula. For each isolated RNA, three repeats of *asp23* expression measurements were performed. For each sample, *asp23* expression level was expressed as percentage change by 100 × [{*asp23* expression_(exposed cells)_ − *asp23* expression_(unexposed control)_}/*asp23* expression_(unexposed control)_].

### Quantification of catalase activity

Catalase activities of the disinfectant-exposed *S. epidermidis* biofilm (n = 62) and planktonic (n = 62) samples and the unexposed controls were determined by a simple visual assay as previously described^52^ with few modifications. Briefly, 100 μL of ~1 × 10^7^ of viable biofilm or planktonic cells/mL was transferred to a Pyrex test tube (1.3 centimetre (cm) diameter by 10 cm height). Then, 100 μL each of 1% Triton X-100 (Rohm and Haas, Philadelphia, USA) and 30% H_2_O_2_ were added, mixed thoroughly and incubated at 25 °C. Once reaction stopped, the height of foam that remained constant for 15 minutes was measured in cm using a ruler. For each sample, three repeats of catalase activity measurements were performed. For each sample, height of foam was expressed as percentage change by 100 × [{height of foam_(exposed cells)_ − height of foam_(unexposed control)_} ⁄ height of foam_(unexposed control)_].

### Statistical analysis

Statistical analysis was performed with GraphPad Prism version 5.03. Data normality was verified by D’Agostino and Pearson omnibus test. Differences between groups were determined using two-tailed paired *t*-test. Data are presented as the mean ± standard error of mean (SEM). A *p*-value less than 0.05 was considered significant.

## Supplementary information

Data set

## Data Availability

All data generated or analyzed during this study are included within this article and its Supplementary Information files.
